# Divergent trajectories of psychological distress during the prolonged COVID-19 pandemic in Japan

**DOI:** 10.1038/s41598-026-53289-7

**Published:** 2026-05-19

**Authors:** Junko Okuyama, Shuji Seto, Takeshi Okuyama, Yu Fukuda, Shinichi Egawa, Fumihiko Imamura

**Affiliations:** 1 Department of Human Health and Nutrition, Shokei Gakuin University, 4-10-1, Yurigaoka, Natori-shi, Miyagi 981-1295 Japan; 2 Faculty of Informatics and Data Science, Akita University, Akita, Japan; 3 Toyota Technological Institute, Nagoya, Japan; 4 Graduate School of Human Sciences, The University of Osaka, Osaka, Japan; 5 International Research Institute of Disaster Science (IRIDeS), Tohoku University, Sendai, Japan

**Keywords:** COVID-19, self-esteem, impostor phenomenon, psychological polarization, psychological resilience, repeated cross-sectional surveys, Japan, Health care, Psychology, Psychology

## Abstract

Psychological responses to the COVID-19 pandemic showed heterogeneous trajectories rather than uniform recovery. We analyzed seven waves of nationwide web-based surveys conducted in Japan between June 2020 and December 2022 (approximately 1,000 participants per wave) to examine long-term trends in depression, anxiety, and stress and their associations with self-evaluative traits. DASS-21 scores were significantly elevated during the initial emergency phase (June 2020) and declined substantially thereafter (Kruskal–Wallis tests, all *p* < .001). However, distributional analyses indicated that a subgroup of individuals remained persistently distressed despite overall population-level improvement. Decision-tree–based variable-importance analyses identified the impostor phenomenon as the strongest contributor to depression, anxiety, and stress, followed by self-esteem. For anxiety, survey timing showed the strongest linear association, whereas non-linear models consistently highlighted self-evaluative variables as dominant predictors. These findings indicate that prolonged societal crises are associated with divergent mental-health adaptation patterns. Psychological self-evaluation factors, particularly impostor feelings and low self-esteem, may play a central role in persistent distress beyond demographic and social-network factors.

**Supplementary Information:**

The online version contains supplementary material available at 10.1038/s41598-026-53289-7.

## Introduction

The COVID-19 pandemic constituted not only a global biomedical emergency but also a sustained psychosocial crisis that significantly altered daily life and mental health^1–3^. In Japan, the initial months of 2020 were characterized by unprecedented uncertainty, stringent behavioral restrictions, and widespread anxiety. The state of emergency in Japan was predicated on non-mandatory behavioral restrictions, often referred to as a “mild lockdown,” which nonetheless resulted in considerable psychological stress^4^. National surveys have documented significant increases in depressive and stress symptoms, reflecting trends observed in other countries^5,6^. However, longitudinal studies suggest that many individuals followed resilient or recovery trajectories over time, gradually regaining relative psychological stability despite repeated waves of infection and ongoing social and economic stressors, including evidence synthesized across pandemic waves showing substantial heterogeneity in stress resilience and mental-health adaptation^1,7–10^.

Nevertheless, psychological recovery from the COVID-19 pandemic has been markedly heterogeneous rather than universal. Longitudinal evidence indicates that, although many individuals showed resilience or gradual improvement, others followed delayed-recovery, persistently distressed, or even deteriorating trajectories over time^10–12^. This uneven pattern of recovery appears to be concentrated among more vulnerable groups, including those with pre-existing mental health conditions^11,12^, suggesting that a subset of the population has remained chronically distressed and unable to return to pre-pandemic levels of well-being. This disparity raises a critical question: What factors determine who recovers and who does not? Identifying the psychological determinants that differentiate resilient individuals from those who remain persistently distressed is crucial for the development of targeted mental health strategies in the context of long-term crises. Recent national data further indicate that psychological distress in Japan has become polarized rather than uniformly improved over time^13^. This interpretation is supported by international longitudinal evidence showing that mental health responses to the COVID-19 pandemic followed heterogeneous trajectories—such as resilient, recovered, chronic, and deteriorating patterns—rather than a single population-wide trend toward recovery^8,10^. Specifically, moderate distress levels decreased during the pandemic, while severe distress increased, particularly among working-age adults, indicating a widening mental health gap^13^. Among the many candidate factors, self-esteem—defined as a global sense of self-worth—has been widely studied as a psychological factor relevant to stress and depression^,^ and has long been regarded as a broad protective resource in mental-health promotion^14^. One influential theoretical account, derived from Terror Management Theory, is the anxiety-buffer hypothesis, which proposes that self-esteem serves as a psychological resource that helps individuals manage existential threat by preserving a sense of self-integrity, meaning, and symbolic security^15,16^. During the early stages of the pandemic, evidence suggested that self-esteem buffered the emotional impact of loneliness and fear under acute uncertainty^17^. Despite extensive research, the role of this factor under prolonged stress exposure remains insufficiently understood, particularly as initially adaptive mechanisms may deteriorate over time. Rather than remaining stable, stress responses can undergo allostatic adaptation, leading to physiological wear-and-tear and altered neuroendocrine regulation^18–21^. As a result, mechanisms that are initially protective may become attenuated or even maladaptive.

Emerging evidence further suggests that prolonged stress does not affect individuals uniformly; instead, it may disproportionately burden those with pre-existing vulnerabilities, thereby widening disparities in emotional outcomes. Consistent with this view, recent studies from East Asia—including network analyses of older adults in Hong Kong—have identified subgroup differences in depressive symptom structure and emotional vulnerability during the pandemic^22^.

Although this factor has been predominantly examined in the context of acute stress, its role under chronic conditions remains less well-defined. Over extended periods, individuals may differ in their capacity to maintain regulatory functioning, rendering resilience-related differences increasingly salient^23,24^. Conversely, the impostor phenomenon—a persistent tendency to doubt one’s competence despite objective evidence of achievement—may function as a vulnerability factor that complicates recovery. Research has consistently linked impostor feelings to poorer mental health outcomes, including psychological distress, burnout, and depressive and anxiety symptoms^25–27^. Individuals with stronger impostor feelings often discount their accomplishments, attribute success to luck, and engage in chronic self-criticism, patterns that may become amplified under prolonged uncertainty and thereby impede psychological recovery. Extensive systematic reviews have shown that impostor feelings are strongly associated with depression, anxiety, and burnout across diverse professional groups^25^. Moreover, research among mental health and other high-responsibility professionals indicates that imposter tendencies predict heightened emotional exhaustion and reduced well-being^28,29^. However, in disaster or pandemic contexts, the available evidence remains methodologically limited, as most studies on healthcare and mental health professionals have been cross-sectional, with relatively little longitudinal follow-up^29–31^.

To address these gaps, the present study analyzed data from seven waves of nationwide web-based surveys conducted in Japan between June 2020 and December 2022, paralleling recent national evidence indicating that psychological distress trajectories became increasingly polarized during the pandemic period^13^. We aimed to elucidate (1) the evolution of psychological distress over the prolonged pandemic period and (2) the influence of self-esteem and impostor feelings on individual differences in psychological distress trajectories. Employing both non-parametric comparisons and machine learning-based predictive modeling, we investigated whether psychological self-evaluation factors were more significant than demographic or persistent psychological distress, consistent with broader theoretical and empirical work on heterogeneity in longitudinal mental health trajectories^32,33^. We hypothesized that (a) depressive symptoms would be highest during the initial phase of the pandemic and decline thereafter, consistent with longitudinal evidence showing that depressive symptoms and broader psychological distress were elevated early in the COVID-19 pandemic and subsequently showed partial improvement^2,7,34^, and (b) low self-esteem would predict sustained psychological distress over time, reflecting long-standing evidence that low self-worth functions as a persistent vulnerability factor for depressive trajectories^35–37^. By identifying these patterns, this study seeks to advance our understanding of how self-concept processes govern long-term psychological adaptation in the face of chronic societal stress.

## Methods

### Study design and setting

**Fig. 1:**
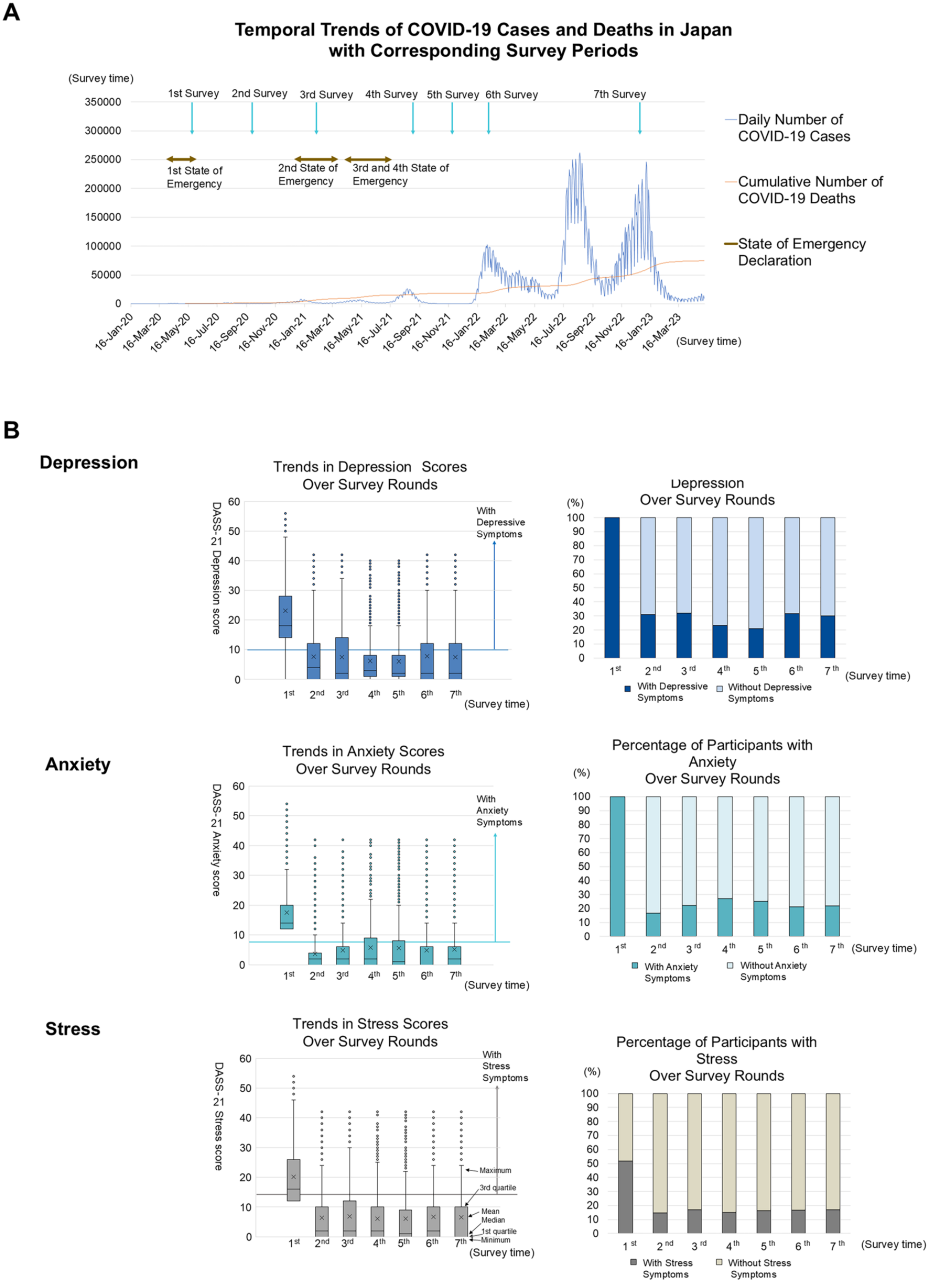
National COVID-19 trends and temporal distribution of psychological distress across seven survey waves in Japan. (**A**) Daily confirmed COVID-19 cases, cumulative deaths, state-of-emergency periods, and timing of the seven survey waves conducted between June 2020 and December 2022. (**B**) Distribution of DASS-21 Depression, Anxiety, and Stress scores across the seven survey waves, shown as box-and-whisker plots, together with the proportion of participants exceeding the clinical cutoff thresholds for each subscale. Clinical cutoffs were defined as depression ≥ 10, anxiety ≥ 8, and stress ≥ 15. Distress levels were highest during the first survey wave and declined substantially thereafter, although a substantial minority remained above the cutoff across later waves.

We conducted seven waves of nationwide web-based surveys in Japan from June 2020 to December 2022. Each wave included a sample of approximately 1,000 adults drawn from extensive online research panels. The inclusion criteria required participants to be aged 18 years or older and to reside in Japan. Participants provided electronic informed consent prior to each wave of data collection. The present analyses concentrated on population-level trends across the waves and examined the roles of self-esteem and the impostor phenomenon in relation to depressive symptoms under prolonged pandemic conditions. The timing of each of the seven surveys is depicted in Fig. 1A, which also illustrates Japan’s national infection trends and periods of emergency declaration. The initial survey was conducted shortly after the first nationwide state of emergency in June 2020, followed by subsequent surveys in September 2020, February 2021, November 2021, February 2022, August 2022, and December 2022. This schedule enabled us to capture both the acute and chronic phases of the COVID-19 pandemic in Japan. During Waves 1 and 2, the distribution reflected the full sample assessed during the acute emergency phase.

### Participants and procedure

Recruitment and data collection were conducted by a professional survey company utilizing stratified invitations to ensure comprehensive representation across age and sex demographics. Each survey wave was administered independently, allowing individuals to participate in multiple waves. All questionnaires were self-administered online. To mitigate response bias, item order was randomized within scales where applicable, and attention-check items were incorporated. The demographic characteristics of participants for each of the seven survey waves are detailed in Table 1. Each wave comprised approximately 1,000 adults, maintaining a balanced sex ratio (1:1) and encompassing a wide age range from 20 to over 65 years. The consistency in sampling across waves facilitated the comparability of psychological indicators over time.

**Table 1:** Demographic characteristics of participants across seven survey waves.

Survey wave	*N* (total)	Female *n* (%)	Mean age (SD)	18–29 *n* (%)	30–49 *n* (%)	50–64 *n* (%)	65 + *n* (%)
1st (Jun 2020)	1,000	500 (50.0)	44.7 (14.1)	180 (18.0)	400 (40.0)	300 (30.0)	120 (12.0)
2nd (Sep 2020)	1,000	498 (49.8)	44.8 (14.0)	172 (17.2)	408 (40.8)	307 (30.7)	113 (11.3)
3rd (Feb 2021)	1,000	501 (50.1)	45.1 (13.9)	165 (16.5)	415 (41.5)	310 (31.0)	110 (11.0)
4th (Nov 2021)	1,000	503 (50.3)	45.0 (14.2)	161 (16.1)	412 (41.2)	309 (30.9)	118 (11.8)
5th (Feb 2022)	1,000	504 (50.4)	44.9 (14.0)	158 (15.8)	414 (41.4)	312 (31.2)	116 (11.6)
6th (Aug 2022)	1,000	499 (49.9)	44.6 (14.3)	164 (16.4)	411 (41.1)	308 (30.8)	117 (11.7)
7th (Dec 2022)	1,000	502 (50.2)	45.2 (14.1)	162 (16.2)	410 (41.0)	310 (31.0)	118 (11.8)

Note: Each wave represents an independent cross-sectional sample (*n* ≈ 1,000) stratified by age and sex (1:1 ratio). All participants were aged ≥ 18 years to simplify the ethical approval procedures.

### Measures

#### Depression Anxiety Stress Scales-21 (DASS-21)

This instrument assesses depressive, anxiety, and stress symptoms using three 7-item subscales rated on a 4-point scale (0–3) based on experiences during the past week^38^. To conform to the conventional scoring range of 0–42, raw subscale scores were doubled. As specified in the DASS manual, severity is classified as normal　(0–9), mild (10–13), moderate (14–20), severe (21–27), and extremely severe (28+)^38^. In the present study, internal consistency across survey waves was excellent for the Depression subscale (Cronbach’s α = 0.941–0.955), good to excellent for Anxiety (α = 0.873–0.932), and excellent for Stress (α = 0.913–0.947 ; Supplementary Table S2).

#### Rosenberg Self-Esteem Scale (RSES)

The Rosenberg Self-Esteem Scale (RSES) is utilized to assess self-esteem through ten items employing a four-point response format^39^. The total score ranges from 10 to 40, with higher scores indicating greater self-esteem. The scale was analyzed as a continuous measure, with tertiles employed for group comparisons, as no universally accepted clinical cutoff exists for Japanese adults. In the present study, internal consistency across the 4th–7th survey waves ranged from acceptable to good (Cronbach’s α = 0.780–0.861 ; Supplementary Table S2).

#### Clance Impostor Phenomenon Scale (CIPS)^40^

This scale evaluates impostor feelings through 20 items, each rated on a scale from 1 (not at all true) to 5 (very true), resulting in total scores ranging from 20 to 100. Higher scores denote stronger impostor cognition. According to the original scoring guidelines, results are categorized as few impostor characteristics (≤ 40), moderate (41–60), frequent (61–80), and intense (≥ 81). In the present study, internal consistency across the 4th–7th survey waves were excellent (Cronbach’s α = 0.940–0.953 ; Supplementary Table S2).

#### Lubben Social Network Scale-6 (LSNS-6)

The scale in question assesses social connectedness through six items, each scored on a scale from 0 to 5, culminating in a total score range of 0 to 30. Higher scores are indicative of more extensive social networks. Within Japanese populations, a score below 12 is commonly employed to identify social isolation^41^. In the present study, internal consistency across survey waves was acceptable to good (Cronbach’s α = 0.861–0.894; Supplementary Table S2).

### Analytical strategy

The analyses were conducted in three distinct phases. Initially, descriptive statistics were employed to encapsulate the characteristics of the participants and the distribution of psychological scales across each wave. Given the non-normal distribution of the psychological variables, medians with interquartile ranges [IQR] were reported instead of means and standard deviations. Subsequently, non-parametric comparisons were utilized to examine differences in depressive symptoms across self-esteem and impostor phenomenon groups, categorized into tertiles based on sample distributions. Kruskal–Wallis tests were applied, followed by post-hoc Dunn tests where applicable; effect sizes are presented as η² for omnibus tests and r for pairwise comparisons. Finally, to assess the relative contributions of psychological, demographic, and social factors to depressive, anxiety, and stress symptoms, generalized regression and decision-tree–based models were fitted. The primary predictive analyses were executed using JMP Student Edition 19.0.1 (SAS Institute Inc., Cary, NC, USA), which employs the same computational engine as JMP Pro for the modeling procedures utilized in this study. Variable-importance indices were derived from decision-tree–based partition models and normalized to ascertain the contribution proportions for each predictor. To verify the robustness of these patterns, tree-based modeling and feature importance estimation were replicated in MATLAB (MathWorks Inc., Natick, MA, USA) using independent scripts for gradient-boosted decision trees and SHAP-based interpretability. The analyses conducted in both JMP and MATLAB produced comparable profiles of predictor influence; therefore, the JMP-based variable importance estimates are presented in Fig. 5B as the primary analytic output.

### Predictive modeling using generalized regression and decision-tree–based variable importance (JMP & MATLAB)

In order to assess the relative contributions of psychological, demographic, and social variables to symptoms of depression, anxiety, and stress, we employed generalized regression modeling and decision-tree–based variable-importance analyses using JMP Student Edition 19.0.1 (SAS Institute Inc., Cary, NC, USA). The Student Edition utilizes the same computational engine as JMP Pro for all modeling procedures applied in this study, including partition modeling and generalized regression. The predictors incorporated were Rosenberg Self-Esteem Scale (RSES) scores, Clance Impostor Phenomenon Scale (CIPS) scores, Lubben Social Network Scale-6 (LSNS-6), age, and survey date (days since the first confirmed COVID-19 case in Japan). Variable-importance values were derived from decision-tree–based partition models and normalized to estimate each predictor’s proportional contribution to the outcome variance. The contribution profiles are depicted in Fig. 5B. To ensure robustness and validate the stability of the variable-importance ranking, we independently replicated the tree-based predictive modeling in MATLAB (MathWorks Inc., Natick, MA, USA). Gradient-boosted decision tree models were trained for each psychological outcome, and the predictor effects were quantified using SHAP-based interpretation. The MATLAB-based results closely mirrored the patterns observed in JMP, confirming that the impostor phenomenon and self-esteem consistently exhibited the strongest contributions across both linear and non-linear modeling frameworks. Collectively, these analyses provide convergent evidence that psychological self-evaluation factors are the predominant determinants of long-term depressive, anxiety, and stress symptoms during the prolonged COVID-19 pandemic.

### Sensitivity and robustness checks

Sensitivity analyses were conducted to explore (a) alternative cut-points for self-esteem and impostor scores, utilizing quartiles instead of tertiles, and (b) models that excluded the LSNS-6 to evaluate the dependence on social connectedness. Additionally, non-parametric tests were repeated using bias-corrected bootstrap confidence intervals (1,000 resamples) to assess the stability of group differences.

### Ethics

All procedures adhered to the ethical standards set forth by both institutional and national research committees, as well as the 1964 Helsinki Declaration and its subsequent amendments. The study protocols received approval from the Institutional Review Board of the International Research Institute of Disaster Science (IRIDeS) at Tohoku University, with approval IDs: 2020-007 on June 15, 2020; 2020-040 on January 15, 2021; and 2021-031 on November 19, 2021. Informed consent was obtained online from all participants prior to their involvement in each survey wave.

### Reporting

This observational study has been reported in accordance with the STROBE guidelines. To enhance reproducibility, a comprehensive analysis plan, variable coding rules (e.g., DASS‑21 subscale scoring, RSES reverse-coded items), and additional robustness checks are included in the Supplementary Materials.

## Results

### Overall trends in psychological symptoms

As shown in Fig. 1A,B, national infection trajectories and emergency declarations corresponded with temporal variation in mental health indicators across the seven survey waves. Depression, anxiety, and stress symptoms were highest during the first survey wave (June 2020), corresponding to the acute phase of the pandemic in Japan, and declined substantially by the second survey wave (September 2020). Thereafter, median symptom levels remained relatively low and stable across subsequent waves.

Median [IQR] DASS-21 depression, anxiety, and stress scores across the seven survey waves are presented in Table 3, and their temporal distributions are shown in Fig. 1B. Kruskal–Wallis tests revealed significant differences across waves for depression (H = 429.65, η² = 0.061), anxiety (H = 477.19, η² = 0.067), and stress (H = 422.70, η² = 0.060), all *p* < .001. These findings indicate significant temporal variation in psychological distress, with the most pronounced shift occurring between the first and second survey waves.

**Table 3:** Median [interquartile range] DASS-21 depression, anxiety, and stress scores across seven survey waves.

Outcome	1st	2nd	3rd	4th	5th	6th	7th	H (df = 6)	*p*	η²
Depression	18 [14–28]	4 [0–12]	2 [ 0–14]	2 [ 0–10]	2 [ 0– 8]	2 [ 0–10]	2 [ 0–10]	429.65	< 0.001	0.061
Anxiety	14 [12–20]	2 [0–4]	2 [ 0– 6]	2 [ 0– 6]	2 [ 0– 6]	2 [ 0– 6]	2 [ 0– 6]	477.19	< 0.001	0.067
Stress	16 [12–26]	2 [0–10]	2 [ 0–12]	2 [ 0– 8]	2 [ 0– 8]	2 [ 0–10]	2 [ 0– 8]	422.70	< 0.001	0.060

Values are presented as median [interquartile range].

DASS-21 = Depression Anxiety Stress Scales-21.

H statistics correspond to Kruskal–Wallis tests across the seven survey waves.

Effect size (η²) was calculated as (H − k + 1) / (N − k), where k = 7 groups.

As shown in Fig. 1B and Supplementary Table S1, although overall median symptom levels declined after the first wave, a substantial minority of participants continued to exceed the DASS-21 clinical cutoff thresholds across later waves. Specifically, from Waves 3–7, clinically elevated symptoms remained present in 21.1–31.9% of participants for depression, 21.4–27.3% for anxiety, and 15.0–17.0% for stress.

These findings suggest that, despite broad population-level improvement, clinically relevant distress persisted in a subgroup of participants throughout the prolonged pandemic period.

### Self-esteem and impostor phenomenon

Data correspond to the values presented in Table 2.

**Table 2:** Median [interquartile range] scores of self-esteem (RSES) and impostor phenomenon (CIPS) across the 4th–7th survey waves.

Survey wave	*n*	RSES total	RSES male	RSES female	p_gender (RSES)	CIPS total	CIPS male	CIPS female	p_gender (CIPS)
4th	1000	25 [23–28]	25 [23–28]	25 [22–28]	0.141	60 [51–65]	60 [51–63]	60 [51–66]	0.081
5th	1000	25 [22–29]	25 [23–29]	25 [21–28]	0.068	60 [49–65]	60 [49–63]	60 [51–69]	< 0.001
6th	1000	25 [22–29]	25 [22–29]	25 [21–28]	0.291	60 [49–65]	60 [49–60]	60 [50–69]	< 0.001
7th	1000	25 [23–29]	26 [24–29]	25 [22–29]	0.033	59 [47–64]	59 [47–60]	60 [49–67]	0.002

Values are shown as median [interquartile range].

RSES = Rosenberg Self-Esteem Scale; CIPS = Clance Impostor Phenomenon Scale.

Sex differences within each survey wave were examined using Mann–Whitney U tests.

No statistically significant overall between-wave differences were observed across the 4th–7th survey waves for either self-esteem or impostor phenomenon (Kruskal–Wallis tests: RSES, H = 6.911, *p* = .075; CIPS, H = 7.087, *p* = .069).

**Fig. 2:**
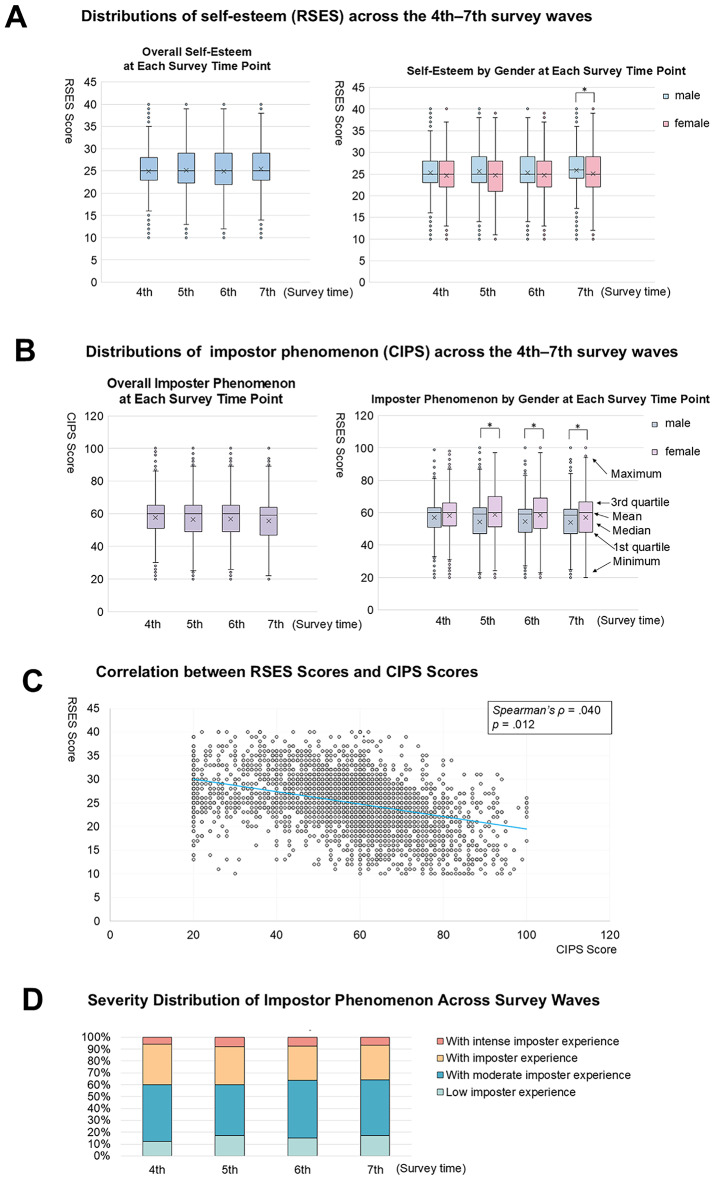
Self-esteem and impostor phenomenon across the 4th–7th survey waves. (**A**) Median [IQR] self-esteem (RSES) scores overall and by sex. (**B**) Median [IQR] impostor phenomenon (CIPS) scores overall and by sex. (**C**) Correlation between RSES and CIPS scores (Spearman’s ρ = − 0.040, *p* = .012).

Self-esteem, as measured by the Rosenberg Self-Esteem Scale (RSES), remained relatively stable across the 4th–7th survey waves (Table 2; Fig. 2A). Median [IQR] scores hovered around 25 [23–29], indicating moderate and consistent self-evaluation over time. Although no significant overall changes were observed (Kruskal–Wallis *p* = .075), sex-based comparisons revealed that males tended to report slightly higher self-esteem than females, reaching significance in the 7th wave (*p* = .033).

In contrast, Clance Impostor Phenomenon Scale (CIPS) scores exhibited a mild but non-significant decline across the same period (Table 2; Fig. 2B). Median [IQR] CIPS values decreased from 60 [51–65] in the 4th wave to 59 [47–64] in the 7th wave. Female participants consistently exhibited higher levels of impostor feelings than male participants, with significant gender differences emerging from the 5th wave onward (*p* < .001–0.002).

**Fig. 3:**
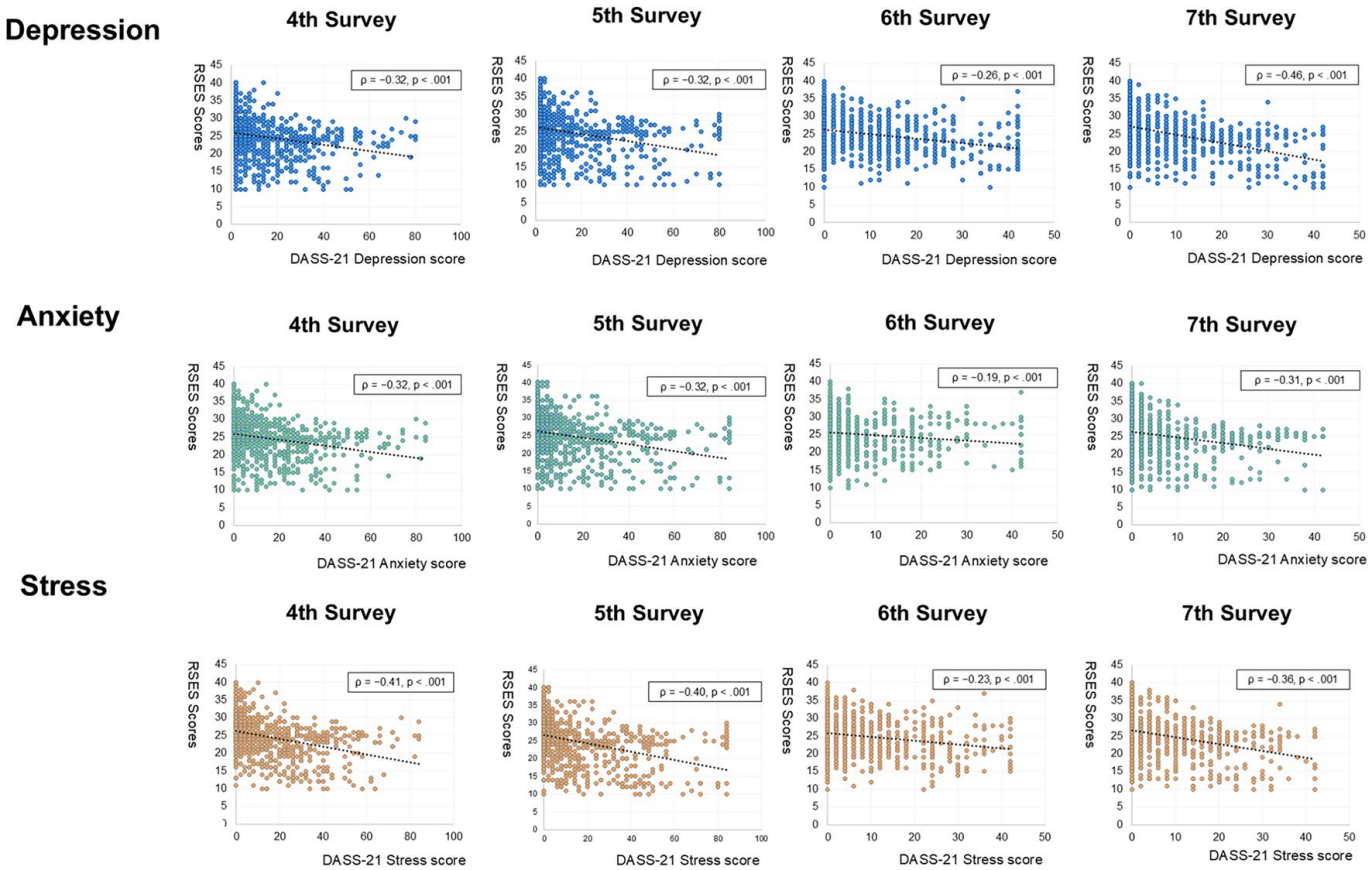
Correlations between self-esteem and psychological distress during the prolonged COVID-19 pandemic. Scatter plots showing the relationships between Rosenberg Self-Esteem Scale (RSES) scores and DASS-21 subscale scores for depression, anxiety, and stress across the 4th–7th surveys. Each panel demonstrates a significant negative correlation (Spearman’s ρ = − 0.26 to − 0.41, *p* < .001), indicating that lower self-esteem is associated with higher levels of depression, anxiety, and stress symptoms.

**Fig. 4:**
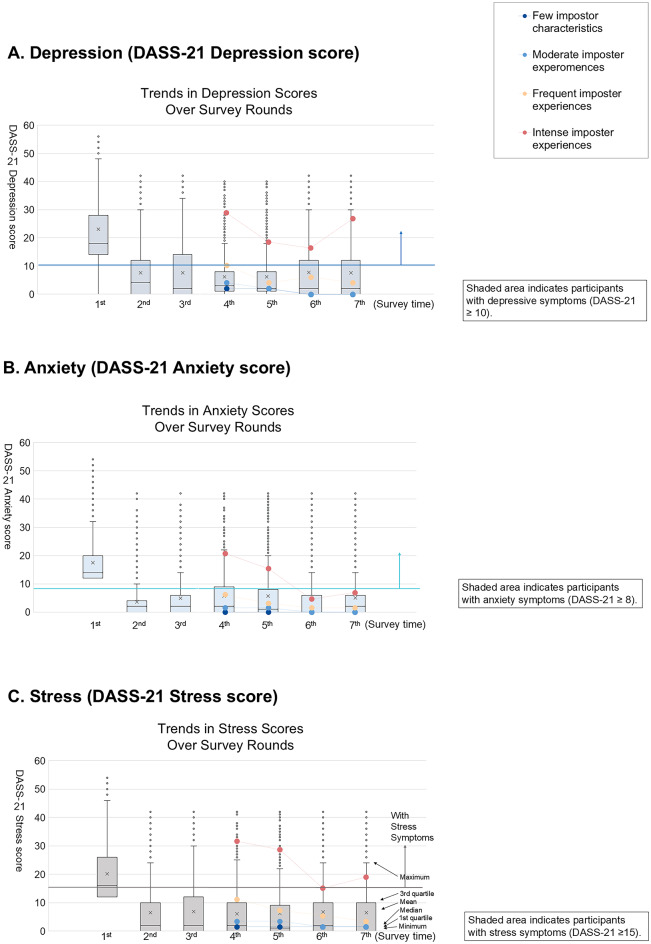
Changes in depression, anxiety, and stress scores across seven survey time points, with symptom distributions stratified by impostor-phenomenon severity (DASS-21). (**A**) Depression, (**B**) Anxiety, and (**C**) Stress scores across the seven survey waves. Box-and-whisker plots represent the distribution of DASS-21 subscale scores at each survey wave. Horizontal reference lines indicate the clinical cutoff thresholds for each subscale (depression ≥ 10, anxiety ≥ 8, stress ≥ 15). Colored points and connecting lines show median symptom scores for participants grouped by Clance Impostor Phenomenon Scale (CIPS) severity categories from the 4th to 7th survey waves: few, moderate, frequent, and intense impostor experiences. Across all outcomes, participants with more severe impostor experiences consistently showed higher levels of psychological distress.

**Fig. 5:**
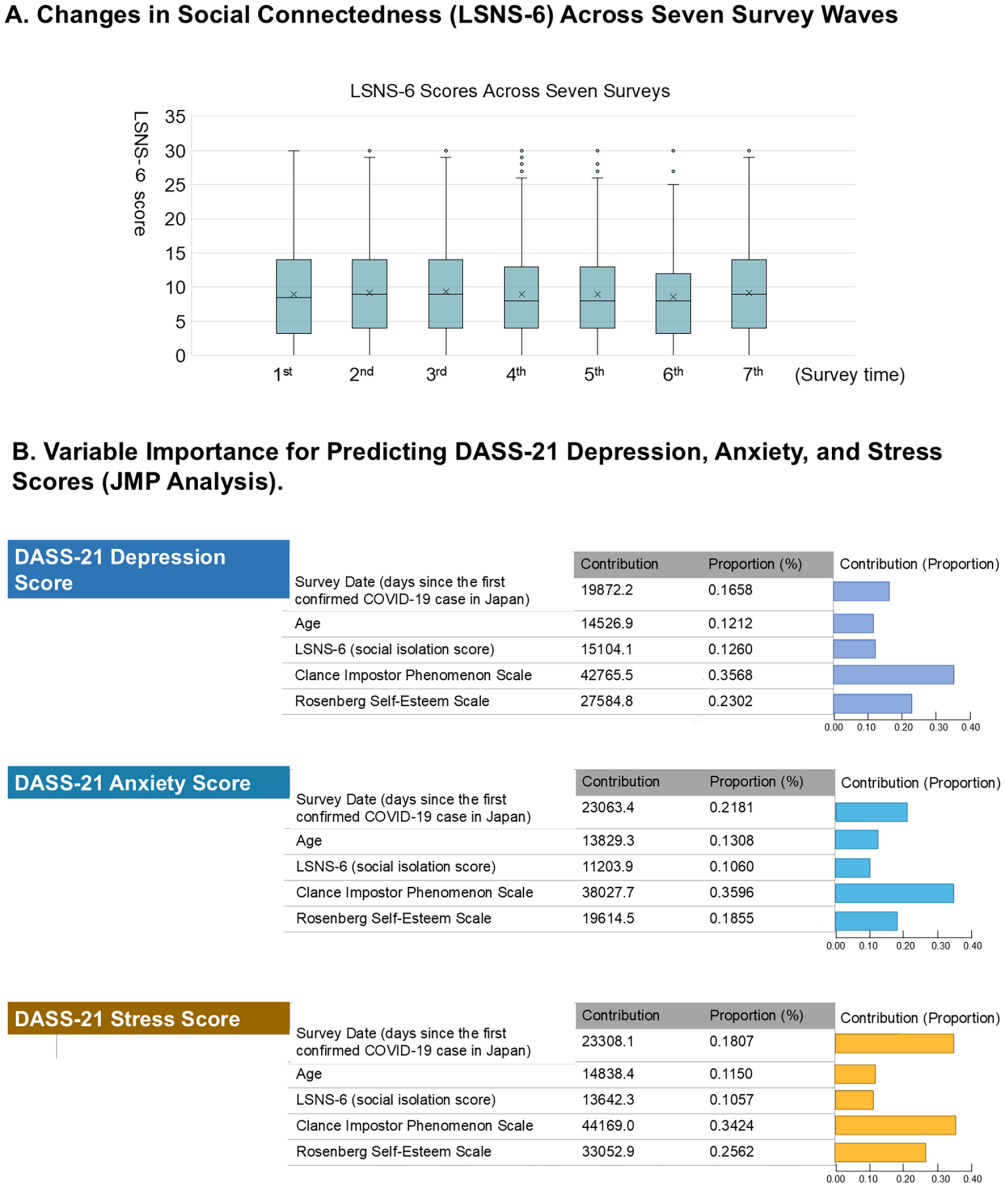
Social connectedness and predictor contributions to psychological distress during the prolonged COVID-19 pandemic. (**A**) Changes in social connectedness (LSNS-6 total scores) across the seven survey waves. No significant differences were observed (Kruskal–Wallis H = 9.54, df = 6, *p* = .145). (**B**) Variable importance for predicting DASS-21 Depression, Anxiety, and Stress scores, based on decision-tree–based contribution proportions (JMP Student Edition 19.0.1). Across all three outcomes, the impostor phenomenon and self-esteem exhibited the largest contributions, followed by the survey date, age, and LSNS-6.

Spearman’s correlation analysis identified a statistically significant but small negative correlation between self-esteem and the impostor phenomenon (ρ = − 0.040, *p* = .012; Fig. 2C). While the effect size was small and unlikely to be clinically meaningful when interpreted as a simple bivariate association, complementary analyses—including group comparisons (Figs. 3 and 4) and variable-importance modeling (Fig. 5B)—consistently indicated that both constructs play substantial and interrelated roles in psychological distress.

### Social connectedness

Social connectedness declined after the initial emergency phase and remained relatively stable thereafter (Table 4; Fig. 5A). Kruskal–Wallis testing indicated no significant differences across waves (H = 9.54, df = 6, *p* = .145), suggesting that subsequent divergence in psychological distress cannot be attributed to major shifts in social network size.

**Table 4:** Median [IQR] scores of social connectedness (LSNS-6) across seven survey waves.

Survey wave	LSNS-6 Median [IQR]
1st (Jun 2020)	14 [9–20]
2nd (Sep 2020)	9 [4–14]
3rd (Feb 2021)	9 [4–14]
4th (Nov 2021)	8 [4–13]
5th (Feb 2022)	8 [4–13]
6th (Aug 2022)	9 [4–14]
7th (Dec 2022)	9 [4–14]

Note: LSNS-6 total score range 0–30; higher values indicate greater social connectedness.

Scores < 12 are often interpreted as indicating social isolation^41^.

Values are presented as medians [25–75th percentiles].

### Group comparisons

As shown in Fig. 3, lower self-esteem was consistently associated with higher levels of depression, anxiety, and stress across the 4th–7th survey waves. Spearman’s rank correlations were significant for all three DASS-21 subscales, indicating that poorer self-evaluation was linked to greater psychological distress.

As shown in Fig. 4, participants with more severe impostor experiences consistently exhibited higher depression, anxiety, and stress scores across the 4th–7th survey waves. In particular, the “intense impostor experience” group remained above or near the clinical cutoff thresholds across outcomes, whereas the “few impostor characteristics” group showed substantially lower symptom levels.

Taken together, these findings indicate that lower self-esteem and more severe impostor experiences were each associated with greater psychological distress during the prolonged pandemic period. To further investigate the relative significance of psychological and social factors in predicting mental health outcomes, we employed variable-importance analyses utilizing JMP’s decision-tree–based modeling framework. Figure 5B illustrates the contribution proportions of five key predictors—impostor phenomenon, self-esteem, social connectedness (LSNS-6), survey date, and age—across depression, anxiety, and stress. Although the generalized regression model demonstrated that the survey date had the strongest linear association with anxiety (Wald χ² = 127.9), the decision-tree-based variable-importance analysis revealed that the impostor phenomenon exerted the largest non-linear contribution to the prediction. This discrepancy highlights the differences in model architecture: linear models capture overall temporal trends, whereas tree-based models are more sensitive to threshold effects and interaction patterns. Collectively, these findings suggest that anxiety during prolonged crises is influenced by both temporal dynamics and self-evaluative vulnerability.

### Predictive modeling

In the variable-importance analysis conducted using JMP (Fig. 5B), the impostor phenomenon and self-esteem emerged as the most significant contributors to symptoms of depression, anxiety, and stress. Specifically, for depressive symptoms, the impostor phenomenon accounted for the largest contribution (proportion = 0.3568), followed by self-esteem (0.2302), survey date (0.1658), age (0.1212), and LSNS-6 (0.1260). In the context of anxiety, the impostor phenomenon again demonstrated the strongest contribution (0.3596), with the survey date ranking second (0.2181), surpassing the influence of self-esteem (0.1855). This finding suggests that anxiety is more susceptible to temporal or situational factors than to other domains. Regarding stress, both the impostor phenomenon (0.3424) and self-esteem (0.2562) were predominant in the contribution profile, while age and LSNS-6 had modest contributions. Collectively, these findings indicate that psychological self-evaluation factors—particularly impostor feelings and self-esteem—are the most influential determinants of long-term mental health status, surpassing demographic and social variables. These findings indicate that psychological polarization during the prolonged pandemic was more strongly explained by self-evaluative vulnerability than by demographic or social-network factors.

## Discussion

Over the course of seven survey waves spanning two and a half years, we observed a clear divergence in the population-level distribution of psychological distress during the prolonged COVID-19 pandemic in Japan. Although depressive symptoms were markedly elevated during the initial emergency phase and declined substantially at the population level thereafter, a stable upper-tail proportion of participants continued to exceed clinical thresholds across later waves. In the present study, polarization refers descriptively to this distributional divergence, characterized by (a) a pronounced decline in median distress levels and (b) the persistence of a clinically elevated subgroup across survey waves.

Notably, despite a median depression score of 2 from Wave 3 onward, between 21% and 32% of participants continued to meet the clinical cutoff (depression ≥ 10). Similarly, clinically elevated symptoms persisted for anxiety (21.4–27.3%) and stress (15.0–17.0%) across Waves 3–7. Kruskal–Wallis tests indicated significant but more modest between-wave differences in overall distress levels, with the most pronounced shift occurring between the first and second survey waves. However, we did not conduct formal dispersion or mixture modeling. Accordingly, polarization is used here to denote coexisting population-level decline and subgroup-level persistence rather than statistically verified bimodality. Given the repeated cross-sectional design, these findings reflect distributional patterns at the population level and do not imply within-person recovery trajectories. Thus, the central question is not whether overall distress decreased, but why recovery became unevenly distributed across the population. This pattern is consistent with recent national evidence suggesting mental-health polarization in Japan^13^.

Our variable-importance analysis conducted using JMP indicated that psychological self-evaluation factors were among the strongest contributors to long-term psychological outcomes. Across the three domains—depression, anxiety, and stress—the impostor phenomenon emerged as the most influential predictor, while self-esteem provided substantial but secondary protection. Notably, anxiety differed from the other domains in that temporal factors (survey date) exerted a stronger influence than self-esteem, suggesting heightened sensitivity to situational and environmental fluctuations. These findings indicate that prolonged stress does not affect all psychological domains uniformly and that persistent self-doubt may represent a particularly potent correlate of chronic distress. The application of multiple modeling frameworks enhanced the robustness and interpretability of our findings by capturing both linear temporal trends and nonlinear psychological mechanisms. Collectively, these findings suggest that self-concept processes may play a central role in long-term psychological adaptation to chronic societal stress. This apparent discrepancy between weak bivariate correlation and strong multivariate contributions may reflect the multidimensional and context-dependent nature of self-evaluative processes under prolonged stress.

### Diverging psychological trajectories in prolonged crises

The initial months of the pandemic reflected an acute stress phase characterized by uncertainty, threat perception, and social isolation^1,2,4^. Over time, however, population-level data indicated partial adaptation and stabilization, consistent with broader longitudinal evidence showing that many individuals followed resilient or recovery-oriented trajectories during the pandemic^7–10^. At the same time, international studies have consistently shown that this recovery was not universal. Rather, mental-health responses to COVID-19 were heterogeneous, with some individuals exhibiting persistent or worsening distress over time^8,10–12,32^.

Our findings align with this literature by suggesting that, as an acute stressor evolves into a prolonged societal condition, individual differences in vulnerability and recovery-related resources become increasingly salient^23,24,31^. In this context, the persistent distress observed among individuals with lower self-esteem may reflect reduced capacity to maintain adaptive self-regulation under chronic uncertainty. Collectively, these findings support the view that prolonged crises may amplify pre-existing disparities in emotional functioning and underscore the need for long-term, stratified mental-health support rather than a uniform post-crisis recovery model.

### Self-esteem as a psychological buffer

Self-esteem consistently emerged as a significant protective factor across all survey waves. In line with the anxiety-buffer hypothesis, higher self-esteem may help individuals tolerate prolonged uncertainty by preserving a sense of self-worth and psychological coherence^15–17^. Individuals with stronger self-esteem may be more likely to appraise ongoing stressors as manageable rather than overwhelming, thereby facilitating emotional recovery even in the absence of major improvements in external conditions.

Conversely, persistently low self-esteem may contribute to a self-reinforcing cycle of negative self-evaluation, hopelessness, and reduced coping confidence^37–39^. This interpretation is consistent with vulnerability models of depression, which suggest that low self-worth is not merely a consequence of distress but may also function as a prospective risk factor for persistent depressive symptoms^37,38^. Our findings extend this perspective by suggesting that the protective role of self-esteem may remain relevant even under prolonged and recurrent societal stress. Interventions that promote self-acceptance, self-compassion, and more adaptive self-appraisal may therefore contribute to longer-term psychological recovery.

### The impostor phenomenon as a compounding vulnerability

The impostor phenomenon, alongside self-esteem, was strongly associated with depressive symptoms, anxiety, and stress. Individuals with stronger impostor feelings—characterized by persistent self-doubt despite evidence of competence—showed consistently higher levels of psychological distress across the later survey waves. This pattern is broadly consistent with previous work linking impostor feelings to psychological distress, burnout, anxiety, and depressive symptoms across diverse populations^25–28^.

In the context of a prolonged societal crisis, impostor cognitions may intensify distress by reinforcing chronic self-criticism, discounting successful coping, and undermining psychological recovery. This interpretation is plausible given prior evidence that impostor feelings are associated with emotional exhaustion and poorer well-being, particularly in high-demand professional contexts^26–28^. At the same time, the pandemic-specific literature on impostor phenomenon remains relatively limited and is still dominated by cross-sectional studies^29,30^. Our findings therefore suggest that maladaptive self-evaluation may represent an important but underexamined vulnerability process in long-duration crises.

### Implications for public mental-health resilience

The identification of divergent adaptation patterns has important implications for public mental-health preparedness in disasters and pandemics. Conventional crisis responses often focus primarily on the acute phase, aiming to reduce immediate fear and uncertainty. However, our findings suggest that the prolonged recovery period may require a different psychological emphasis—namely, the preservation of self-worth, adaptive self-evaluation, and sustained coping capacity. Community-based and digital interventions designed to strengthen these capacities may help reduce the risk of chronic distress in vulnerable subgroups.

In addition, incorporating psychological self-evaluation indicators into repeated mental-health surveillance may improve the early identification of individuals at elevated risk for persistent distress. The present findings also illustrate the value of combining conventional statistical approaches with machine-learning models to capture both broad temporal patterns and non-linear vulnerability structures. Such approaches may prove useful for identifying subgroups that are not fully visible through average population trends alone.

#### Limitations and future directions

This study has several limitations. First, all measures were self-reported and may therefore be subject to reporting bias, although the scales used in this study demonstrated acceptable to excellent internal consistency and included established Japanese versions where available^38–41^. Second, although repeated cross-sectional surveys enabled broad population coverage across multiple pandemic phases, they do not allow direct inference regarding within-person change or causal direction. Third, while the Japanese version of the Clance Impostor Phenomenon Scale was developed with permission and expert guidance, formal psychometric validation remains ongoing.

In addition, our models did not include several contextual variables that may influence long-term adaptation, such as income, occupation, prior psychiatric history, or direct COVID-19 infection experiences. Future research would benefit from integrating longitudinal cohort designs, broader psychosocial covariates, and multi-method assessments—including physiological or neurocognitive markers—to better clarify how self-evaluative processes interact with chronic stress regulation over time. Cross-cultural comparisons will also be important for determining whether the patterns observed here generalize beyond Japan or reflect sociocultural tendencies related to modesty, self-criticism, and interpersonal evaluation.

## Conclusion

Prolonged societal crises do not produce uniform psychological consequences; rather, they reshape the population distribution of distress. In Japan’s extended COVID-19 pandemic, overall median symptoms declined after the acute phase, yet a clinically elevated subgroup persisted across survey waves, reflecting distributional divergence at the population level. This pattern was more strongly associated with self-evaluative vulnerability—particularly impostor phenomenon and low self-esteem—than with demographic or social-network factors.

These findings suggest that chronic societal stress may amplify latent differences in self-concept, resulting in uneven adaptation trajectories within the same social context. Strengthening psychological self-worth and addressing maladaptive self-evaluation processes may therefore represent key components of long-term mental-health preparedness in future protracted crises.

## Supplementary Information

Below is the link to the electronic supplementary material.

Supplementary Material 1

Supplementary Material 2

Supplementary Material 3

Supplementary Material 4

## Data Availability

The datasets generated and/or analysed during the current study are not publicly available due to ethical restrictions and the sensitive nature of the mental health data, but are available from the corresponding author on reasonable request.
