# Supplementary figures and images for: Divergent trajectories of psychological distress during the prolonged COVID-19 pandemic in Japan
Source: Sci Rep. 2026 May 19;16:22745. doi: 10.1038/s41598-026-53289-7 (PMC13385922; doi:10.1038/s41598-026-53289-7)

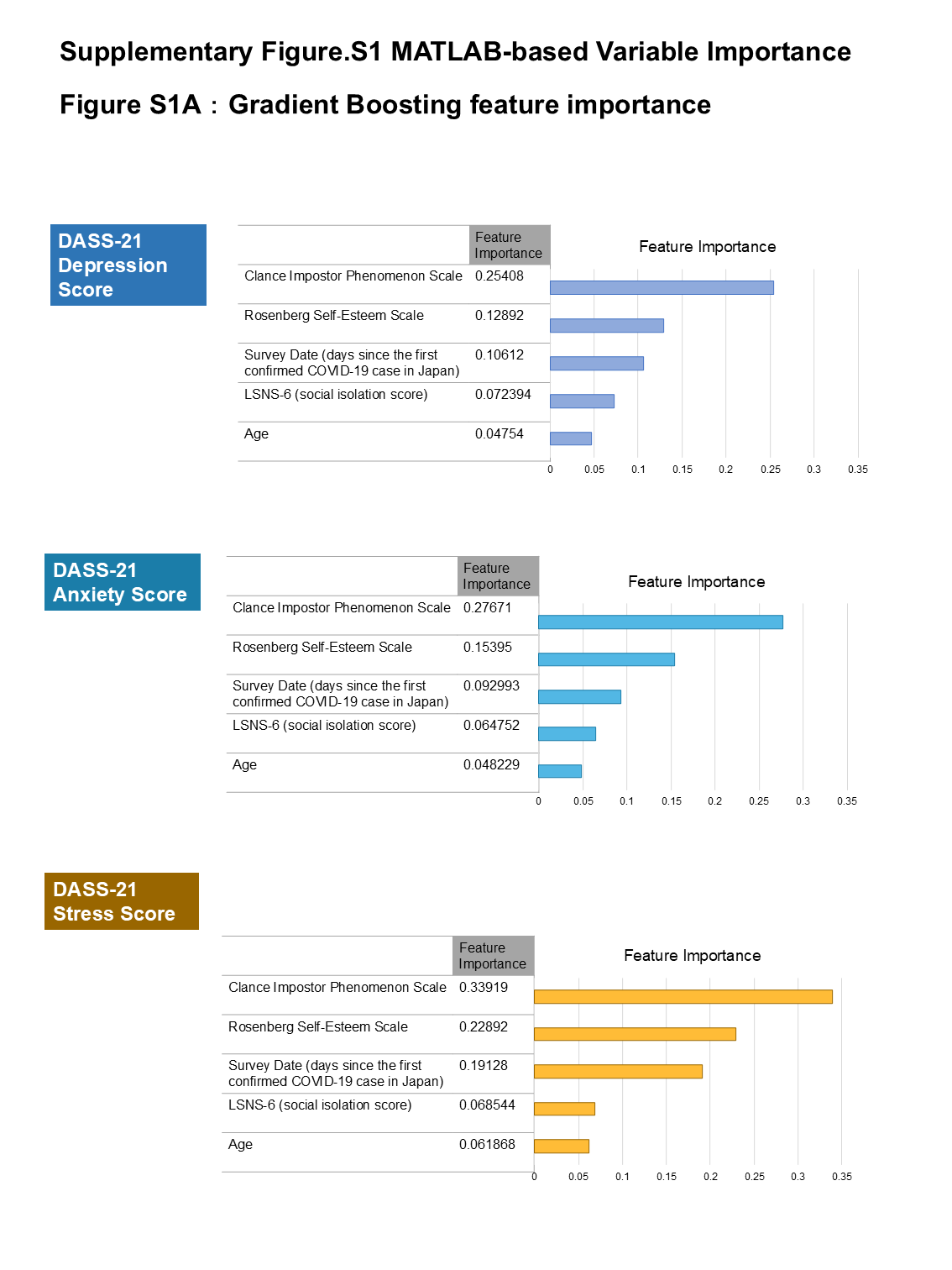

Supplement: Supplementary file 1 — Supplementary Material 1 [file 41598_2026_53289_MOESM1_ESM.png]

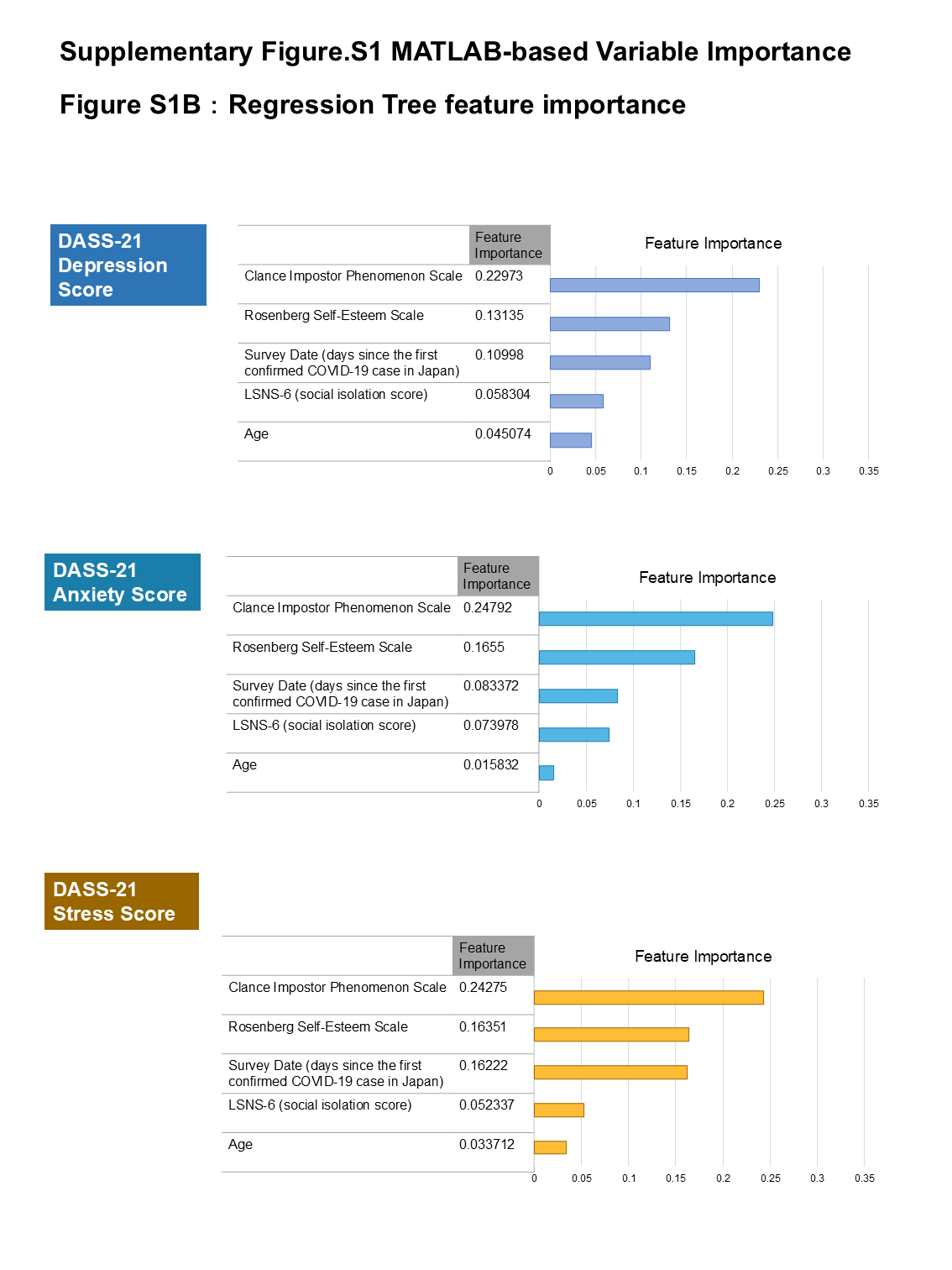

Supplement: Supplementary file 2 — Supplementary Material 2 [file 41598_2026_53289_MOESM2_ESM.png]
